# Black-capped chickadees (*Poecile atricapillus*) alter alarm call duration and peak frequency in response to traffic noise

**DOI:** 10.1371/journal.pone.0241035

**Published:** 2020-10-29

**Authors:** Jason R. Courter, Rebecca J. Perruci, Kelsey J. McGinnis, Jacqueline K. Rainieri

**Affiliations:** Department of Natural Science, Malone University, Canton, Ohio, United States of America; University of Missouri Columbia, UNITED STATES

## Abstract

Anthropogenic noise is an often-overlooked byproduct of urbanization and affects the soundscape in which birds communicate. Previous studies assessing the impact of traffic noise have focused on bird song, with many studies demonstrating the ability of birds to raise song frequency in the presence of low-frequency traffic noise to avoid masking. Less is known about the impact of traffic noise on avian alarm calls, which is surprising given the degree to which predator information within alarm calls may impact fitness. The objective of this study was to assess the impacts of traffic noise on the Black-capped Chickadees (*Poecile atricapillus*), a small non-migratory songbird with a well-studied and information-rich alarm call. We studied birds at eight locations in Stark County, Ohio, from 15 January to 7 March 2016, and used a taxidermic mount of an Eastern Screech-Owl to elicit alarm calls. In half of the trials, a pre-recorded traffic noise track was also broadcasted at 50 decibels. In noise trials, chickadee calls contained more introductory notes (P < 0.001), more total notes (P < 0.001), were of longer duration (P < 0.001), and had lower introductory and D-note peak frequencies (P = 0.032 and P = 0.041, respectively). No differences were noted in the number of D-notes per call between noise and control trials. Modifying alarm call duration and frequency, without changing the number of D-notes, may be a strategy that chickadees use to convey predator information and to coordinate a threat-appropriate mobbing response when it is not possible to change call type. Our results add to the small, but growing, literature documenting the effects of anthropogenic noise on avian alarm calls, demonstrate the flexibility and complexity of chickadee calls given in response to predators, and may partially explain why chickadees adapt well to urban areas.

## Introduction

Anthropogenic noise is an often overlooked byproduct of urbanization [1, 2], and nearly one-fifth of the land area of the United States is exposed to traffic noise [3–5]. Such noise can prevent animals from conveying acoustical information [2, 5] and is particularly problematic for birds that rely on sound to defend territories, attract mates, and alert flockmates to the presence of predators [2, 6, 7].

Many birds use low-frequency songs and calls to transmit information over long distances and against environmental obstructions because higher frequency sounds attenuate faster [2]. Traffic noise usually occupies lower frequency ranges [8, 9], and overlap between human and animal noise can lower the threshold at which birds are able to detect and discriminate between vocalizations (i.e., ‘masking’) [2, 3, 10] and appropriately respond [7, 11, 12]. To combat this issue, birds can: shift vocalization frequencies (i.e., ‘the acoustic adaptation hypothesis’); reviewed by Roca et al. [13], increase the amplitude of vocalizations (i.e., ‘the Lombard Effect’) [7, 14], shift calling to times of the day when anthropogenic noise is less prevalent [15], repeat portions of their vocalizations [16], and alter the duration of vocalizations [17, 18].

Previous studies assessing the impacts of anthropogenic noise on avian vocalizations have focused primarily on song [3, 11], with some studies comparing conspecific songs in quiet and noisy areas to assess evolutionary adaptation [19–21] and others broadcasting experimental noise in quiet areas to assess behavioral plasticity [10, 12, 17]. A commonly reported finding is that birds can adjust their song frequencies to reduce masking and improve information transmission [10, 17, 19, 20, 22].

While the focus on understanding the impacts of anthropogenic noise on bird song is well-justified, it is surprising how little is known about the impacts of noise on bird calls, particularly given the role of calls in flock coordination and predator defense [2, 3, 11]. Recent studies indicate that Spotted Doves (*Spilopelia chinensis*) raise the minimum frequencies of their cooing vocalization in more urban areas [23] and Noisy Miners (*Manorina melanocephala*) shift their low-frequency call-notes upward in urban areas [24]. Conversely, Billings [25] reported that minimum frequencies of alarm calls in three families of birds were lower in urban areas, proposing that low-frequency alarm calls may be less susceptible to masking amid urban soundscapes.

Black-capped Chickadees (*Poecile atricapillus*) inhabit deciduous and coniferous woodlands and urban areas throughout the northern United States and Canada [26]. Both their songs and alarm calls exhibit marked complexity [27, 28], with ‘fee-bee’ songs consisting of two pure alternating notes in the 3–4 kHz range [32], and broadband ‘chick-a-dee’ calls consisting of short, high-frequency introductory notes (i.e., ‘A’, ‘B’, and ‘C’ notes) in the 4–9 kHz range [29] and longer, low-frequency D-notes in 3–6 kHz range [27, 29, 30]. Songs are primarily given during the breeding season, whereas ‘chick-a-dee’ calls are given throughout the year to convey predator information, coordinate flock movement, and convey food information [27, 31]. Alarm call notes are generally given in order, but calls are otherwise flexible, and notes can be repeated, omitted, or acoustically altered in different contexts [29, 31, 32].

Black-capped Chickadees quickly increase the frequency of their ‘fee-bee’ songs in response to anthropogenic noise [33], particularly when the noise overlaps with dominant song frequencies [12]. Males in noisy areas also sing higher-frequency songs than males in quiet areas [34]. To date, however, it is unclear how anthropogenic noise affects alarm calls of Black-capped Chickadees. Grace and Anderson [9] reported that the frequency parameters of D-notes in similar Carolina Chickadee (*Poecile carolinensis*) alarm calls did not change in response to a traffic noise gradient. Jung et al. [35] also reported that Carolina Chickadees responded similarly to a Screech-Owl playback in environments of varying noise levels, potentially indicating that noise was not interfering with information transfer. On the other hand, when Black-capped Chickadees were exposed to similar alarm calls of Tufted Titmice (*Baeolophus bicolor*), mobbing decreased in noise trials, suggesting that important alarm call information may have been masked [36]. To further understand the possible impacts of anthropogenic noise on the alarm calls of Black-capped Chickadees, we compared the structural and acoustical properties of chickadee alarm calls given in the presence and absence of broadcasted traffic noise to assess whether alarm calls could be altered to prevent masking.

## Methods

Black-capped Chickadees were studied at eight locations in Stark County, Ohio, from 15 January 2016 to 7 March 2016. Field sites were selected based on accessibility and the likelihood that chickadees would be present and consisted of suburban residences (N = 3), county parks (N = 3), a nature center (N = 1), and a private farm (N = 1). If an active feeder was not already established at a site, we hung a tube feeder and kept it stocked with black-oil sunflower seeds for the duration of our study to reliably attract birds.

Field sites were located >800 m from each other to minimize the chance of recording the same birds at different locations [9] and trials were conducted between 0800–1600 each day, with at least 48 hours between trials to prevent habituation. During trials, we placed a taxidermic mount of an Eastern Screech-Owl (*Megascops asio*) on a platform 1 m away from the bird feeder to elicit alarm calls [31, 37, 38]. We initially covered the Screech-Owl for a 5-minute acclimation period [39] before removing the covering and recording chickadee vocalizations for an 8-minute trial period using a Roland R-26 Omni-directional Portable Field Recorder at a distance of 5 m.

Each site was visited at least twice, with one trial consisting of only the owl presentation to elicit ‘normal’ alarm calling behavior (i.e., our control group), and the other consisting of the owl presentation in conjunction with traffic noise (i.e., our experimental group) that was broadcast 5 meters from the feeding station at the beginning of the trial. The traffic noise track (see sample here: http://soundbible.com/641-Urban-Traffic.html#Urban%20Traffic%20Sound) was 2:30 minutes in length and repeated on a continuous loop during noise trials through an iHome Portable Speaker at an amplitude of 50 decibels. It was assembled to approximate a variety of noises that birds might encounter in an urban environment [15] and consisted of continuous low-frequency traffic noise between 0–2 kHz, approximately 30 intermittent instances of broadband traffic noise associated with cars passing that ranged from 2–12 kHz, and occasional short, high frequency noises between 10–12 kHz associated with horn beeps and car brakes. A field observer recorded chickadee calls given in field trials from a distance of 5 m using a Roland R-26 Omni-directional Portable Field Recorder.

**Fig 1:**
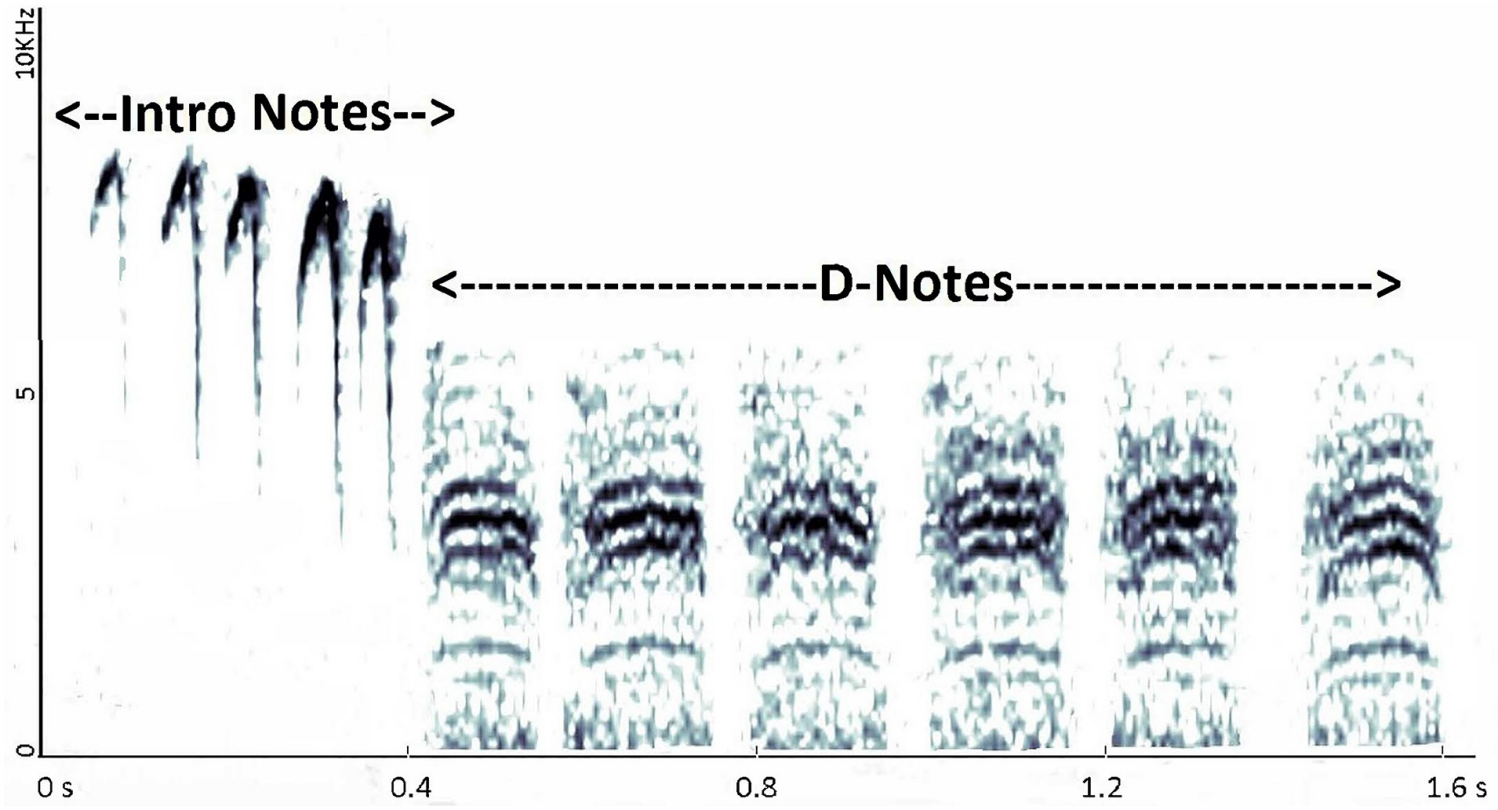
A typical chickadee alarm call consisting of introductory notes and D-notes. Figure spans a duration of 1.6 seconds (x-axis) and a frequency range of 0–10 kHz (y-axis). Peak frequency values of introductory and D-notes were identified by Raven as the location of each note type where amplitude was the highest (i.e., the darkest in our figure).

We used Raven Pro Version 1.5 (Cornell Lab of Ornithology, Ithaca, NY, U.S.A.) to display sonograms of alarm call recordings and identify the first 20 alarm calls in each trial to capture a period that chickadees perceived as high-threat [20, 38]. Calls were identified as sounds between 0–13 kHz consisting of at least two discernable notes and free of indistinguishable overlap from other calls. We counted the number of ‘introductory notes’ in each call (i.e., ‘A’, ‘B’ and ‘C’) and measured their total durations [29, 37, 40]. We also identified the number and total duration of ‘D’ notes (Fig 1). Minimum frequency measurements were difficult to ascertain, particularly in noise trials where they overlapped with low-frequency traffic noise [41–43], and thus we recorded peak frequencies (i.e., the frequency of the highest amplitude within the given call element) [9, 13, 21, 22]. When analyzing sonograms, care was given to ensure that peak frequency measurements were from alarm calls and not from traffic noise [43]. We used t-tests (JMP, Version 14.0 SAS Institute Inc., Cary, NC) to compare the mean number of introductory notes per call in each trial, the mean number of D-notes per call in each trial, the mean duration of introductory notes per call in each trial, the mean duration of D-notes per call in each trial, and the mean peak frequencies of introductory notes and D-notes in each trial.

## Results

In total, we recorded alarm calls in eight experimental trials (i.e., calls given in response to an Eastern Screech-Owl in the presence of traffic noise) and in seven control trials (i.e., calls given in response to the Screech-Owl only). We analyzed the first 20 usable alarm calls in each trial, if present, and this resulted in 145 alarm calls in our experimental group and 113 alarm calls in our control group. Despite our repeated efforts, we were unable to record a usable control trial at one of our study sites.

Chickadees uttered alarm calls with more introductory notes per call ($\bar{\text{x}}=4.24\pm 2.76$ S.D. vs. $\bar{\text{x}}=2.72\pm 2.58$) and more total notes per call ($\bar{\text{x}}=8.50\pm 3.84$ S.D. vs. $\bar{\text{x}}=6.44\pm 3.57$ S.D.) when traffic noise was present compared to when traffic noise was absent (P < 0.001 and P < 0.001; Table 1). The mean duration of introductory notes in calls was longer in noise trials ($\bar{\text{x}}=0.42\;\text{s}\pm 0.27$ vs. $\bar{\text{x}}=0.35\;\text{s}\pm 0.21$; P = 0.027), as was the total alarm call length when D-notes were included ($\bar{\text{x}}=1.24\;\text{s}\pm 0.64$ in noise trials vs. $\bar{\text{x}}=0.99\;\text{s}\pm 0.62$ in control trials; P < 0.001). When comparing acoustical elements of introductory notes, chickadees uttered calls with lower peak frequencies in noise trials ($\bar{\text{x}}=6704.4\;\text{Hz}\pm 762.1$ vs. $\bar{\text{x}}=6955.8\;\text{Hz}\pm 871.1$; P = 0.032; Table 1).

**Table 1:** Chickadee alarm call components compared in noise and control trials using t-tests.

	Noise Trials^a^		Control Trials^b^			
Call Component	Mean	S.D.	Mean	S.D.	t-stat	P-value
*Introductory Notes*						
Number	4.24	2.76	2.72	2.58	4.57	< 0.001
Duration (s)	0.42	0.27	0.35	0.21	2.23	0.027
Peak Frequency (Hz)	6704.4	762.1	6955.8	871.1	-2.16	0.032
*D-Notes*						
Number	4.26	2.85	3.73	2.9	1.47	0.14
Duration (s)	0.95	0.55	0.94	0.52	0.15	0.88
Peak Frequency (Hz)	3646.6	179.7	3700.8	192.4	-2.06	0.041
*Total Notes*						
Number	8.5	3.84	6.44	3.57	4.44	< 0.001
Duration (s)	1.24	0.64	0.99	0.62	3.15	< 0.001

^a^ N = 145 calls analyzed.

^b^ N = 113 calls analyzed.

No differences were noted in the number of D-notes per call in noise trials when compared to control trials ($\bar{\text{x}}=4.26\pm 2.85$ vs. $\bar{\text{x}}=3.73\pm 2.90$; P = 0.14), or in the total duration of all D-notes in alarm calls ($\bar{\text{x}}=0.95\;\text{s}\pm 0.55$ vs. $\bar{\text{x}}=0.94\pm 0.52$; P = 0.88; Table 1). D-notes uttered in noise trials had lower peak frequencies ($\bar{\text{x}}=3646.6\;\text{Hz}\pm 179.7$) than calls uttered in control trials ($\bar{\text{x}}=3700.8\;\text{Hz}\pm 192.4$; P = 0.041; Table 1).

## Discussion

Our results (Table 1) demonstrate a high degree of flexibility in the alarm calling of Black-capped Chickadees whereby call duration and acoustical structure are altered in the presence of traffic noise. In noise trials, chickadees gave calls that contained more introductory notes, more total notes, alarm calls of longer duration, and alarm calls with notes of lower peak frequencies (Table 1).

### Comparing our control treatment to previous studies

Alarm calls from our control group (i.e., recorded in response to Screech-Owl only) were similar in composition to what we had expected based on previous studies. For example, we report $\bar{\text{x}}=2.72\pm 2.58$ introductory notes per call in our control group compared to Baker and Becker [44] who reported 3.7 introductory notes per call when exposing Black-capped chickadees to a taxidermic mount of a Prairie Falcon (*Falco mexicanus*). Although we noted more D-notes per call in our control group than Baker and Becker [44] (6.44 ± 3.57 S.D vs. 2.7 notes per call), this is not surprising given that Prairie Falcon body length is approximately double that of Eastern Screech-Owl [44, 45] and body length corresponds to the degree of perceived threat by chickadees [31]. The high number of D-notes that we report is in line with what Soard and Ritchison [37] reported (i.e., 7 D-notes per call) when exposing Carolina Chickadees to a taxidermic mount of an Eastern Screech-Owl.

### Comparing notes per call and alarm call duration in noise and control trials

Chickadees uttered calls with 6.44 (± 3.57 S.D.) notes per call and a mean duration of 0.99 s (± 0.62 S.D.) in controls trials, while uttering calls with 8.50 (± 3.84 S.D.) notes per call and a mean duration of 1.24 s (± 0.64 S.D.) in noise trials (Table 1). This difference was driven largely by a greater number of introductory notes in noise trials (4.24 notes per call vs. 2.72 notes per call; P < 0.001) and not a difference in number of D-notes per call (4.26 notes per call vs. 3.73 notes per call P = 0.14; Table 1). A difference in number of introductory notes and total alarm call duration, without notable differences in number of D-notes, may indicate that chickadees did not view the Screech-Owl as more threatening in our noise trial, per se, particularly because changing the number of D-notes may incorrectly convey information to flockmates about predator threat level [31]. Instead, adding introductory notes may have ensured that most notes dodged the masking effects of intermittent higher-frequency traffic noise and were still able to coordinate a threat-appropriate mobbing response [27, 40].

Many studies, but not all [22, 41, 46], report that birds lengthen their vocalizations in noisy environments. For example, Hill et al. [47] reported that Oriental Magpie-Robins (*Copsychus saularis*) in urban areas sang longer songs and Walters et al. [21] showed that Northern Mockingbirds (*Mimus polyglottos*) sang songs with more syllables in unmasked frequency regions in urban areas. Halfwerk and Slabbekoorn [17] found that Great Tits (*Parus major*) sang certain songs longer in noisy conditions, particularly songs with frequency ranges unmasked by noise. In the presence of noise, birds may add elements to their vocalizations or repeat vocalizations to ensure that information is accurately conveyed [8]. In general, bird calls are less plastic and variable than bird songs, which further supports the need for other call properties, such as duration, to be altered to communicate effectively in noisy conditions. If this is the case, then longer calls could indicate that a bird is well-adapted to its environment and could even be a measure of social dominance in non-breeding contexts, just as longer songs can signal male quality and fitness in the breeding season [48]. Lengthening song or speech to ensure that information is accurately conveyed in noisy conditions (also referred to as ‘temporal auditory summation’ and described by Owens [8]) has also been observed in primates (summarized by Brumm et al. [49]), and cetaceans [50]. Our results differ from those of Grace and Anderson [9] who reported no differences in duration or notes per call in Carolina Chickadees in response to urban noise, although they measured differences along a naturally occurring noise gradient and recorded alarm calls without predator models. Francis et al. [51] remind us that marked differences in acoustical responses to noise may exist between taxonomically similar species.

### Comparing alarm call frequencies in noise and control trials

Chickadees lowered the peak frequencies of their alarm call elements in noise trials (Table 1). To our knowledge, we are the first to document this change in Black-capped Chickadee alarm calls in response to traffic noise, but if the main purpose is to avoid masking, we expect that the adjustments that we note are similar to adaptive adjustments noted in chickadee songs. Previous studies [12, 34, 52] have shown that Black-capped chickadees raise the frequencies of their songs in response to traffic noise to overcome the effects of masking, and this trend has been reported for other species as well [21, 53, 41]. Halfwerk and Slabbekoorn [17] showed that Great Tits (also in Family Paridae) raised the frequency of their songs when exposed to low-frequency noise, but lowered frequency in response to high frequency noise. Given the acoustically heterogeneous traffic noise that we presented in noise trials with intermittent high frequency and broadband sounds, lowering the peak frequencies of introductory and D-notes (Table 1) may have helped chickadees better transmit predator information. Billings [25] proposed that low-frequency alarm calls may be less susceptible to masking in urban habitats and that selective pressures shaping bird songs that are learned may be different than selective pressures shaping alarm calls that are innate [54]. Furthermore, LaZerte et al. [34] reported that chickadees in quieter areas sang songs at lower frequencies in noise trials than in urban environments. They proposed that the potential frequency extremes present in the dawn chorus in quieter areas could make a downward shift in frequency adaptive to prevent interspecific masking. Low-frequency notes may also attenuate less [34] and may be able to be uttered for longer from a physiological standpoint [55].

Contrary to our findings, Grace and Anderson [9] reported that Carolina Chickadees did not alter the frequencies of the D-notes in their alarm calls along an urban to rural gradient, although they did not measure changes in introductory note frequency. Grace and Anderson [9] concluded that given the broadband nature of chickadee calls, flock identify could potentially be maintained by relying on unmasked overtones or the relative spacing of overtones present in alarm call notes as opposed to the frequency measures that we report in our study. We also assessed the impacts of broadcasted traffic noise in otherwise quiet areas in response to a taxidermic mount of a Screech-Owl, whereas Grace and Anderson [9] recorded Carolina chickadee alarm calls along an urban-rural gradient in a more opportunistic fashion, perhaps making it less likely that calls were given in strict predator defense contexts. LaZerte et al. [34] investigated whether Black-capped Chickadee adjustment to noise was dependent on previous exposure and found that males in noisy areas sang higher-frequency songs than males in quiet areas, but only males accustomed to long-term noise were able to immediately adjust frequencies in response to noise. This suggests that chickadees may need time to learn how to adjust to noisier areas, and given this finding, the differences that we note between control and noise trials (Table 1) may have been even more pronounced if we had observed birds that were already adapted to urban environments.

### Study distinctive and future areas of research

Our results add to the small, but growing, literature [9, 23–25, 56] documenting the effects of anthropogenic noise on bird calls. To our knowledge, we are the first to use a taxidermic mount of a predator to elicit alarm calls, which helped ensure that calls were given in predator defense contexts, which strengthens and contextualizes our findings. We also utilized a heterogeneous noise track, which may have better simulated the traffic noise that birds would have encountered in an urban area [15], but at the same time offered a less controlled measure of tonal frequencies in noise trials [12, 17, 57]. Our study also assessed short-term response of wild birds to noise in otherwise quiet areas, a measure of behavioral plasticity, similar to [12, 17, 58], whereas studies that compare birds already adapted to rural and urban environments [22, 24, 34], or along urban-rural gradients [9, 59], may be better positioned to explain evolutionary changes [8, 60]. Future studies should also assess how effectively modified alarm calls of Black-capped Chickadees transmit information in noisy environments and whether they elicit appropriate flock responses (for similar examples, see [7, 36, 61, 62]). Future studies may also assess the ability of chickadees to response to noise of varying amplitude and in variable threat contexts [31, 37, 38]. Taken together, our results further demonstrate the remarkable complexity of the chickadee alarm call. Modifying call duration and frequency may be strategies that Black-capped Chickadees use to communicate effectively in noisy areas when it is not possible to change call type and may partially explain their ability to adapt in urban areas.

## Supporting information

S1 Data (XLSX)
